# Discovery of a DNA methylation episignature for Weiss-Kruszka syndrome

**DOI:** 10.1007/s00439-026-02846-1

**Published:** 2026-07-20

**Authors:** Haley McConkey, Liselot van der Laan, Sourav Ghosh, Lotte Kleinendorst, Michael A. Levy, Jessica Rzasa, Johanna M. van Hagen, Quinten Waisfisz, Heidi L. Schulz, Corina Heller, Kerstin Huhn, Carolin D. Obermaier, Konrad Platzer, Rami Abou Jamra, Nikos Marinakis, Danai Veltra, Konstantina Kosma, Christalena Sofocleous, Peter Henneman, Bekim Sadikovic, Mieke M. van Haelst

**Affiliations:** 1 Verspeeten Clinical Genome Centre, London Health Science Centre, London, ON Canada; 2 Department of Pathology and Laboratory Medicine, Western University, London, ON Canada; 3 Department of Human Genetics, Amsterdam UMC, Amsterdam, The Netherlands; 4 Amsterdam Reproduction and Development, Amsterdam University Medical Centers, University of Amsterdam, Amsterdam, The Netherlands; 5 Emma Center for Personalized Medicine, Amsterdam UMC, Amsterdam, The Netherlands; 6 Zentrum für Humangenetik Tübingen, Tübingen, Germany; 7 Institute of Human Genetics, University of Leipzig Medical Center, Leipzig, Germany; 8 Laboratory of Medical Genetics, Medical School, National and Kapodistrian University of Athens, “Aghia Sophia” Children’s Hospital, Athens, Greece; 9 Research University institute for the study and prevention of genetic and malignant disease of childhood, Athens, Greece; 10 Laboratory of Genetics, Faculty of Medicine, Democritus University of Thrace, Alexandroupolis, Greece

## Abstract

Weiss-Kruszka syndrome (WSKA; OMIM 618619) is a rare autosomal dominant neurodevelopmental disorder caused by haploinsufficiency of ZNF462, a zinc-finger transcription factor involved in chromatin regulation and early embryonic development. WSKA is characterized by developmental delay, hypotonia, craniofacial dysmorphic features (around 8) and variable congenital anomalies. Genome-wide DNAm profiling was performed on peripheral blood DNA from 9 WSKA cases with (likely) pathogenic *ZNF462* variants and matched controls to look for differential methylation. Analysis using the EpiSign™ pipeline identified a robust DNAm pattern, or episignature, specific to WSKA syndrome. Supervised machine-learning classification demonstrated high sensitivity and specificity, with reproducibility confirmed by leave-one-out cross-validation, as well as correctly classifying a validation case with a pathogenic *ZNF462* variant. Comparative analysis revealed partial overlap of genome-wide DNA methylation changes between the WSKA episignature and other neurodevelopmental disorders involving chromatin regulators. Functional annotation of differentially methylated probes and regions demonstrated enrichment for pathways related to neurodevelopment, neuron function and cell adhesion. These findings define and validate a distinct DNAm episignature for WSKA, providing a valuable diagnostic biomarker to support variant classification and offering insight into the epigenomic consequences of ZNF462 haploinsufficiency.

**Supplementary Information:**

The online version contains supplementary material available at 10.1007/s00439-026-02846-1.

## Introduction

*ZNF462* (located at chromosome 9q31.2) encodes a C2H2-type zinc-finger transcription factor. Heterozygous loss-of-function variants in *ZNF462 *cause Weiss-Kruszka syndrome (WSKA; OMIM: 618619), a rare autosomal-dominant disorder (Hau et al. 2025; Weiss et al. 2017). The majority of previously reported pathogenic variants are truncating variants, including nonsense and frameshift supporting haploinsufficiency as the primary disease mechanism (van der Laan et al. 2024). ZNF462 plays a critical role in embryonic development and chromatin regulation. It has been shown in model systems that the murine homolog, Zfp462, is required during early embryogenesis for neural lineage specification by directing heterochromatin formation at enhancers of non-neural (mesodermal/endodermal) genes. Loss of Zfp462 leads to aberrant activation of these non-neural programs during neural differentiation (Yelagandula et al. 2023). This finding provides a mechanistic basis of pathogenicity suggesting that haploinsufficiency of *ZNF462* may disrupt chromatin architecture and cell-fate specification, which could manifest as altered epigenetic marks in humans.

Clinically, WSKA is characterized by a recognizable constellation of features, though with considerable phenotypic heterogeneity. Common findings include neurodevelopmental impairment (global developmental delay, motor/speech delay), hypotonia, feeding difficulties, and in some cases structural anomalies of the brain (such as corpus callosum dysgenesis), heart defects, growth retardation or short stature, and other congenital anomalies. The majoriy of cases present with craniofacial dysmorphic feature such as ptosis, down-slanting palpebral fissures, arched eyebrows, metopic ridging/craniosynostosis, a short upturned nose with bulbous tip, prominent Cupid’s bow, and ear anomalies. More recently, additional growth hormone deficiency and endocrine abnormalities have been reported in some patients (Hau et al. 2025; Weiss et al. 2017; van der Laan et al. 2024; Constantinou et al. 2023; Brady et al. 2023; González-Tarancón et al. 2022; Zhou et al. 2022; Park et al. 2021; Kruszka et al. 2019). Considering that*ZNF462* plays a key role in chromatin remodeling and early neurodevelopment, these clinical features suggest that epigenetic dysregulation may represent an underexplored mechanism contributing to WSKA.

DNA methylation (DNAm) is an essential epigenetic mechanism regulating gene expression and maintaining cellular identity. Pathogenic variants in genes that encode chromatin regulators or transcription factors frequently lead to altered methylation landscapes (Bjornsson 2015; Kerkhof et al. 2022). In a variety of neurodevelopmental disorders, including those caused by chromatin-related genes, disorder-specific DNAm patterns, called ‘episignatures’, have been identified and successfully applied as diagnostic biomarkers, aiding in variant classification and syndrome diagnosis (Sadikovic et al. 2020, 2019; Aref-Eshghi et al. 2018). EpiSign™ is a novel research-use only software that uses genome-wide DNAm analysis and machine-learning algorithms to determine if an individual displays a disorder-specific episignature using peripheral blood DNA (Kerkhof et al. 2024). Reporting and interpretation recommendations for DNAm episignature analysis were recently reported. In 2399 cases assessed by EpiSign, positivity was 18.7% for a broad screen of > 120 disorders, and 32.4% for targeted assessments of suspected disorders (Kerkhof et al. 2024). To increase diagnostic yield, the number of detectable episignatures are currently expanded, especially for conditions where the causative gene is a part of the epigenetic machinery.

Given the function of ZNF462 in chromatin regulation, its role in early neuronal lineage commitment, and the broad, variable clinical phenotype seen in WSKA, we hypothesize that there are impacts to the methylome that may present as an episignature. Identification and validation of such an episignature could provide diagnostic support for WSKA, especially in less severely affected cases not presenting with the striking features of WSKA or in individuals with gene variants of uncertain significance (VUS). This can provide insight in epigenomic consequences of *ZNF462* haploinsufficiency, as well as contribute to our understanding of how perturbations in chromatin regulation translate into neurodevelopmental and congenital phenotypes.

In this study, we aim to define and validate a DNAm episignature associated with WSKA using individuals with confirmed pathogenic *ZNF462* variants and compare genome-wide DNAm profiles between patients with WSKA and those with other neurodevelopmental disorders for which there is a known episignature.

## Methods

### Subjects and study cohort

In this study, we investigated a cohort of nine individuals carrying *ZNF462 *variants identified through diagnostic targeted exome sequencing (ES)(ID exome panel). The cohort consisted of six males and three females. Variant interpretation was performed according to the ACMG/AMP guidelines (Riggs et al. 2020; Abou Tayoun et al. 2018; Richards et al. 2015). The majority of identified *ZNF462* variants (7/9) were predicted loss-of-function alleles, consistent with the established disease mechanism of *ZNF462* haploinsufficiency.

Within the discovery cohort (*n* = 7), we identified four nonsense variants, one canonical splice-site variant, and one frameshift deletion. One additional individual carrying a missense variant classified as a variant of uncertain significance (VUS) was included as a test case for episignature assessment. The validation cohort (*n* = 1) included a single frameshift duplication, classified as likely pathogenic.

### Methylation data processing and episignature analysis

DNA was extracted from the peripheral blood of the cohort participants who present with a WSKA phenotype and carry a pathogenic *ZNF462 *variant, as well as a participant with a VUS and a validation case with a pathogenic variant. Genome-wide DNAm data was generated using the Infinium methylation EPIC Bead Chip array (Illumnia, San Diego, CA) on bisulfite converted genomic DNA according to the manufacturer’s protocol. The intensity data files (iDATs) containing the DNAm signal at each probe were then preprocessed and normalized using the SeSAMe package (version 1.22.2) (Zhou et al. 2018).

The methylation data was analyzed using a standard pipeline, as previously described (Levy et al. 2022), in R (version 4.5.1). Briefly, the DNAm data for the following probes were excluded to ensure that any detected DNAm differences are related to WSKA disease status: 1) probes that did not meet the initial detection p-value threshold of < 0.1, those that overlapped with single-nucleotide variants, those who were cross-reactive and probes present on the X or Y chromosomes. Probes with beta values of 0 and the upper 1% most variable (variance) probes within the case or control samples were excluded. Finally, any probes not included on both EPIC v1 and EPIC v2 Illumina arrays were also excluded.

A set of controls was selected from the EpiSign™ Knowledge Database (EKD) (Kerkhof et al. 2024)from healthy individuals and other patient groups based on sex, age, batch and array type (EPIC v1 and v2) using the R software package MatchIt (version 4.5.2) (Ho et al. 2011). For each WSKA case sample, eight matched controls were assigned for each (case: control ratio of 1:8), leading to the selection of 56 control samples. Data structure was examined using PCA to address any potential technical batch or array effects, as well as to remove any control outliers. Methylation levels (beta values) were converted into M-values using logit transformation, and the transformed values were utilized for linear regression modeling with the limma package (version 3.54.2) (Ritchie et al. 2015). Estimated blood cell proportions (using the Houseman method) (Houseman et al. 2012) were added to the model matrix as confounding variables. The generated p values were moderated using the eBayes function and we used the Benjamini-Hochberg procedure to control for multiple testing by adjusting p-values to control the false discovery rate (FDR). Probes that had a mean methylation difference of less than 5% between the case and control samples were removed.

Parameters for probe selection were adjusted to improve the differentiation between the case and control samples as previously described (Levy et al. 2022). Assessment of case and control separation was completed using hierarchical clustering, specifically Ward’s method on Euclidean distance, and multidimensional scaling (MDS), which involved scaling the pairwise Euclidean distances between samples. The set of probes that best separated case and controls were selected as WSKA episignature probes. To evaluate reproducibility of the episignature, leave-one-out sample cross-validation was performed for each sample in the WSKA cohort and evaluated using hierarchical clustering, MDS, and methylation variant pathogenicity (MVP) plots (described below).

To assess sensitivity and specificity of the episignature, the e1071 R package version 1.7–4 was used to train a support vector machine (SVM) and for the construction of a multiclass prediction model as previously described (Levy et al. 2022; Aref-Eshghi et al. 2021). The WSKA episignature was trained against all remaining controls and other episignature cohorts in the EKD (Kerkhof et al. 2024). 75% of EKD samples were used for training and 25% were used for testing. This was repeated four times so that each EKD sample was used at least once for testing (4-fold training/testing cross-validation). A final classifier for each cohort was made by training case samples against all EKD samples to generate the final SVM classifier. SVM decision values were converted to probability scores according to Platt’s scaling method, which were then used to create the MVP plots. The MVP score predicts the probability that a sample’s methylation pattern matches a given episignature, with scores closest to one indicating the highest probability.

### VUS assessment and validation of newly defined episignature

To test the episignature identified using our WSKA cohort, we tested two samples, Case 8 and Case 9. Case 8 carries a *ZNF462* VUS and presents with features that are not suggestive of WSKA and Case 9 carries a likely pathogenic variant in *ZNF462 *and presents with WSKA features. DNA methylation data was generated as previously described above, and the data was assessed for concordance with the WSKA episignature (Kerkhof et al. 2024). Briefly, the case data was assessed using the SVM classifier, to generate MVP scores for the newly defined WSKA episignature. The cases were also assessed using unsupervised clustering, specifically Euclidean hierarchical and multidimensional scaling (MDS) clustering, to determine if the cases cluster with WSKA cohort or the controls. Cases that match the WSKA episignature exhibit an elevated MVP score as well as concordant unsupervised clustering with WSKA cases.

### Functional annotation and comparative analysis

The functional correlation between the genome-wide DNAm changes and other neurodevelopmental disorders included in the EpiSign™ V5 was performed based on previously published studies (Levy et al. 2022; Silva et al. 2025; Haghshenas et al. 2024, 2024; van der Laan et al. 2025). Briefly, the percentage of all differentially methylated probes (DMPs), not only the probes selected for episignature development, shared between the WSKA cohort and the other neurodevelopmental disorders on the EpiSign™ v5 classifier (Kerkhof et al. 2024) were calculated and visualized using heatmaps generated with R package pheatmap (version 1.0.12) as represented in Fig. 2 (Gu et al. 2014), and circos plots generated with R package circulize (version 0.4.15), as represented in Fig. 3 (Gu et al. 2014). Comparison of the global mean methylation differences between syndromes with known episignatures, and WSKA, was compared in Fig. 5.

Similarities of all differentially methylated probes between the episignature cohorts, including the WSKA cohort, were analyzed using agglomerative clustering as previously described (Levy et al. 2022). The median methylation level of each DMP was computed across all samples with the same condition, forming a beta value matrix, followed by computing Euclidean distances between cohorts and clustering these distances using Ward’s method. For this analysis, we focused on the top 500 DMPs for each cohort, ranked by their p-values. If a cohort had fewer than 500 DMPs, all available DMPs were included in the analysis. The results were visualized as a tree-and-leaf diagram using the R package TreeAndLeaf version 1.6.1 (Cardoso et al. 2022) to incorporate additional information such as global mean methylation difference and total number of DMPs identified for each cohort.

Using identified DMPs for the WSKA cohort, a gene set analysis was performed to investigate the enrichment of Gene Ontology (GO) terms. This analysis was executed using the R packages msigdbr (version 7.5.1) and missMethyl (version 1.36.0). Finally, DMRcate (version 3.0.5) (Peters et al. 2015) identified differentially methylated regions (DMR) with ≥ 5 CpGs within 1 kb of each other. Minimum absolute mean methylation difference between cases and controls was set to 0.1 and significant results selected using a Fisher’s p value cut-off of 0.01. GO analysis was also completed using identified DMRs.

Genomic annotation of DMRs was performed using the R/Bioconductor package ChIPseeker (v1.44.0). DMR coordinates were mapped to the hg19 (GRCh37) human reference genome using the UCSC Known Genes database (TxDb.Hsapiens.UCSC.hg19.knownGene, version 3.2.2). Promoter regions were defined as the window spanning 3 kilobases (kb) upstream and downstream of the Transcription Start Site (TSS). DMRs were categorized based on their overlap with known genomic features, including promoters, exons, introns, and intergenic regions. For each DMR, the distance to the nearest TSS was calculated; regions with a distance of 0 classified as directly overlapping the TSS. Gene symbol assignment was facilitated using the org.Hs.eg.db database (version 3.21.0) to provide functional context for the nearest associated transcripts.

## Results

### Molecular characterization of the cohort

The molecular details of our cohort are summarized in Table 1. All individuals carried a variant in the *ZNF462* gene. The individuals listed as “Discovery” cohort type (case 1 to 7) possess a pathogenic *ZNF462 *variant and received a clinical diagnosis of WSKA syndrome. An additional case (Zhou et al. 2022) who carried a VUS was used to test the episignature, and finally case 9 is a participant who has a likely pathogenic variant and a diagnosis of WSKA syndrome and was used to validate the new episignature. Case 1, 2 and 9 are previously described (van der Laan et al. 2024) and the other cases are publicly available on ClinVar.

**Table 1:** ZNF462 variants detected in our participants NM_021224.4

Case	Gender	Cohort type	Variant	Variant type	Classification
1	Male	Discovery	c.4792 A > T, (p.Lys1598*)	Nonsense	Pathogenic
2	Female	Discovery	c.4792 A > T, (p.Lys1598*)	Nonsense	Pathogenic
3	Male	Discovery	c.2283G > A, (p.Trp761*)	Nonsense	Pathogenic
4	Female	Discovery	c.6829 C > T, (p.Arg2277Ter)	Nonsense	Pathogenic
5	Male	Discovery	c.220 + 1G > A, (p.?)	Splice site	Pathogenic
6	Female	Discovery	c.3700 C > T, (p.Arg1234Ter)	Nonsense	Pathogenic
7	Male	Discovery	c.5531_5544del, (p.Leu1844Hisfs*9)	Frameshift	Pathogenic
8	Male	Test	c.5114 C > G, (p.Thr1705Ser)	Missense	VUS
9	Male	Validation	c.3078dup, (p.Val1027Cysfs*5)	Frameshift	Likely pathogenic

*VUS* variant of uncertain significance

### Investigation and validation of WSKA episignature

**Fig. 1:**
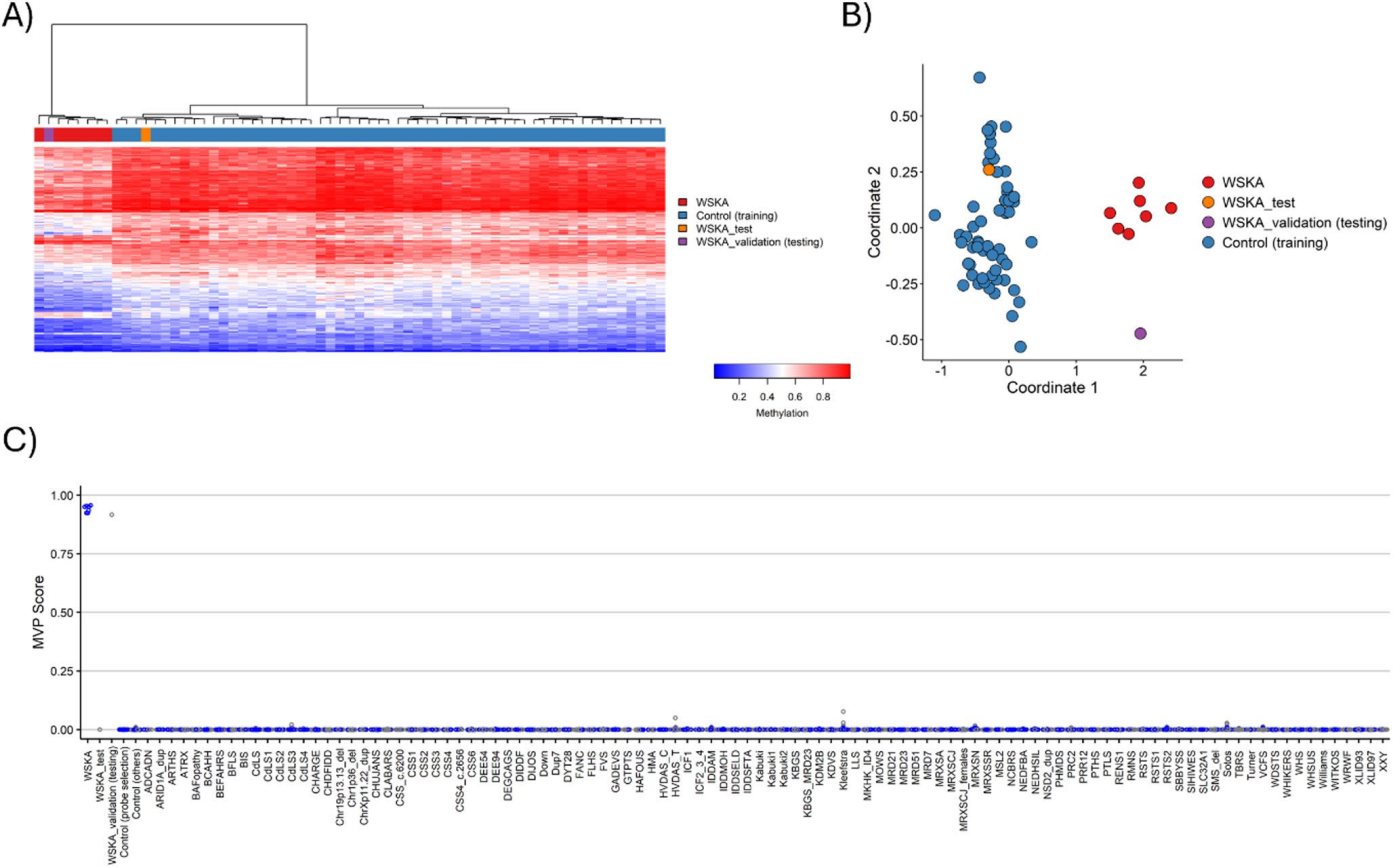
Episignature discovery and validation.** A** The Euclidean clustering plot displays the discovery/training WSKA cases used to develop the episignature as the red data points, with the likely pathogenic validation case in purple, the VUS testing case in orange, and the matched controls as blue points. The rows represent the selected probes for the identified episignature, while the columns represent the training cases and controls. Methylation levels are color-coded, with blue indicating 0 beta values and red indicating 1 beta values. The colour intensity reflects the intensity values of the methylation levels. The validation case demonstrates DNAm changes similar to the WSKA cases and the testing (VUS) sample demonstrates a DNAm profile similar to controls. **B** The Multidimensional Scaling (MDS) plot visualizes similarity of two data points based on distance, with less distance between points indicating more similarity. WSKA cases (red) cluster together and away from matched controls (blue). The validation case (purple), with the likely pathogenic *ZNF462* variant, clusters more closely with WSKA cases whereas the testing case with the *ZNF462* VUS clusters with the controls. **C** The Support Vector Machine (SVM) classifier, trained using identified WSKA episignature probes as features, predicts the probability of a case matches the WSKA episignature. The model was trained using the WSKA cohort, their matched controls, all other unaffected controls, as well as samples from the EKD that were used to develop other condition episignatures. A leave-25%-out cross-validation approach was employed. The average Methylation Variant Pathogenicity (MVP) score was computed and plotted for both investigational/test samples (gray) and training samples (blue). The validation case and the testing (VUS) case were tested against the SVM classifier, with the validation case demonstrating strong concordance with the WSKA episignature (0.9 MVP) and the testing case showed no concordance (0.00019 MVP)

Episignature analysis using 7 WSKA training/discovery cases for probe selection, identified 200 differentially methylated probes, with the majority (88.5%) hypomethylated in WSKA cases (Supplemental Table 1). These probes robustly separated WSKA training cases from age- and sex-matched controls in both MDS and Euclidean hierarchal clustering (Fig. 1A and B). Leave-one-out cross validation demonstrated reproducibility of a WSKA episignature, as all left out WSKA cohort cases consistently clustered with remaining training cases and demonstrated an elevated MVP score (Supplemental Fig. 1). Left out WSKA cases with lower MVP scores (Cases 3, 4 and 5) demonstrated strong unsupervised clustering in both the heatmaps and MDS plots, indicating concordance with other training cases. The SVM classifier was created using the identified 200 episignature probes and trained against all other controls and episignature cohort cases in the EKD (Fig. 1C). The SVM classifier demonstrates a specific and sensitive episignature, with all WSKA training cases demonstrating high MVPs and all other controls and episignature cohorts showing very low or zero MVP scores when used as testing samples. We used a validation sample, Case 9, to determine if our WSKA episignature was sensitive to a new case (not used in episignature training/discovery) carrying a likely pathogenic variant and presenting with features in-keeping with WSKA. The case, purple in Fig. 1A and B, clusters with other WSKA cases used to develop the episignature (red) in both Euclidean clustering heatmap and MDS plot. The case also demonstrated an MVP score of 0.9, showing strong concordance with the newly identified WSKA episignature (Fig. 1C).

### VUS assessment using validated WSKA episignature

One main use for disorder-specific episignatures is to aid in the interpretation of variants of uncertain significance (VUS). Our cohort includes a testing case, Case 8, a patient who carries a VUS in *ZNF462* however, the clinical presentation is in line with WSKA. When testing Case 8 against our WSKA episignature (orange case in Fig. 1A and B), it does not cluster with cases WSKA training cases in the Euclidean hierarchal clustering heatmap or MDS plots, showing similarity instead to controls. The case also demonstrated a very low MVP score (Fig. 1C). These findings suggest that Case 8’s VUS is likely benign; however, episignature assessment alone is not sufficient to fully rule out pathogenicity, especially for variant types such as missense, which may lead to atypical phenotypes or distinct episignatures.

### Functional correlation of WSKA episignature with other disorders included in EpiSign^™^ V5 classifier

**Fig. 2:**
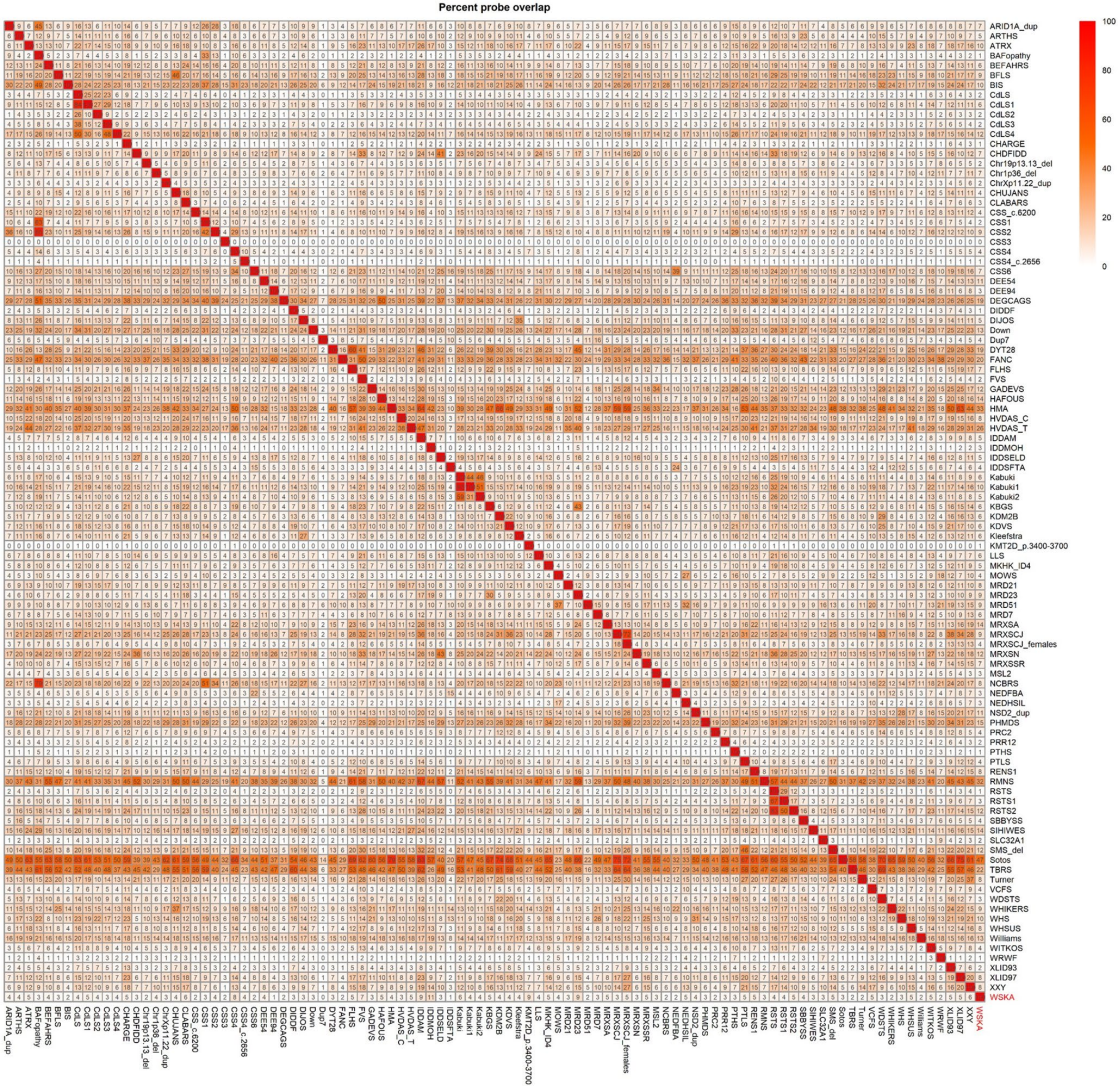
Percentage of shared global differentially methylated probes between WSKA and episignature disorders. The heatmap plot illustrates the proportion of shared probes between each pair of cohorts. The color scale represents the percentage of DMPs of the cohort represented on the y-axis that are also present in the cohort represented on the x-axis

**Fig. 3:**
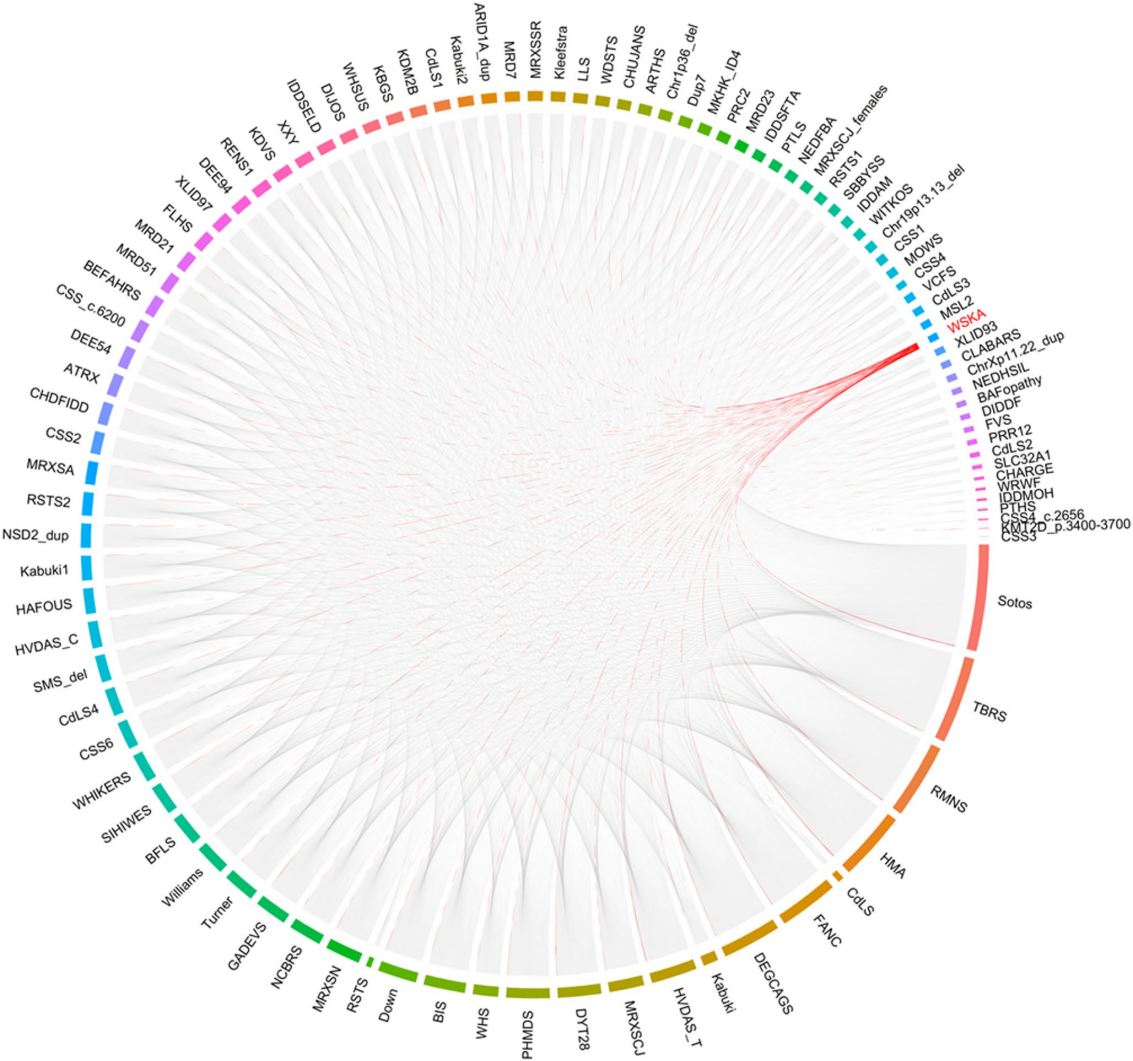
Visualization of differentially methylated probes in WSKA with other cohorts with known episignatures. The circos plot is utilized to depict the shared DMPs between the WSKA cohort and other episignature cohorts. The thickness of the connecting lines within the plot corresponds to the number of probes shared between the WKSA cohort and other conditions, offering a visual representation of the degree of probe overlap

We compared the genome-wide methylation changes in our WSKA cohort with all other disorder cohorts that have a defined episignature on EpiSign™ v5, as previously described (Kerkhof et al. 2024; Levy et al. 2022). Visualization of the percentage of all overlapping probes that are differentially methylated (DMPs) in multiple conditions, including WSKA, is summarized in Fig. 2. Pairwise comparisons were made between all conditions and the colors within each square of the heatmap indicate the percentage of the bottom cohort’s probes that are also found in the right cohort’s probes. The WSKA cohort demonstrated most overlap with Sotos syndrome (OMIM #117550; 47% overlap) caused by (likely) pathogenic variants in histone methyltransferase *NSD1*, Rahman syndrome (OMIM #617537, RMNS; 32% overlap) caused by (likely) pathogenic variants in linker histone protein *HIST1H1E*, Hunter-McAlpine craniosynostosis syndrome (OMIM# 601379, HMA; 30% overlap) caused by Chr5q35-qter duplications that include *NSD1*, and Helsmoortel-van der Aa syndrome, specifically cases where the (likely) pathogenic variants occurs in the N- and C-terminus of transcription factor *ADNP* (OMIM# 615873, HVDAS_T; 26% overlap). Specific overlap of all DMPs identified in the WSKA cohort (3632 DMPs) with the other episignature cohorts is represented in Fig. 3.

**Fig. 4:**
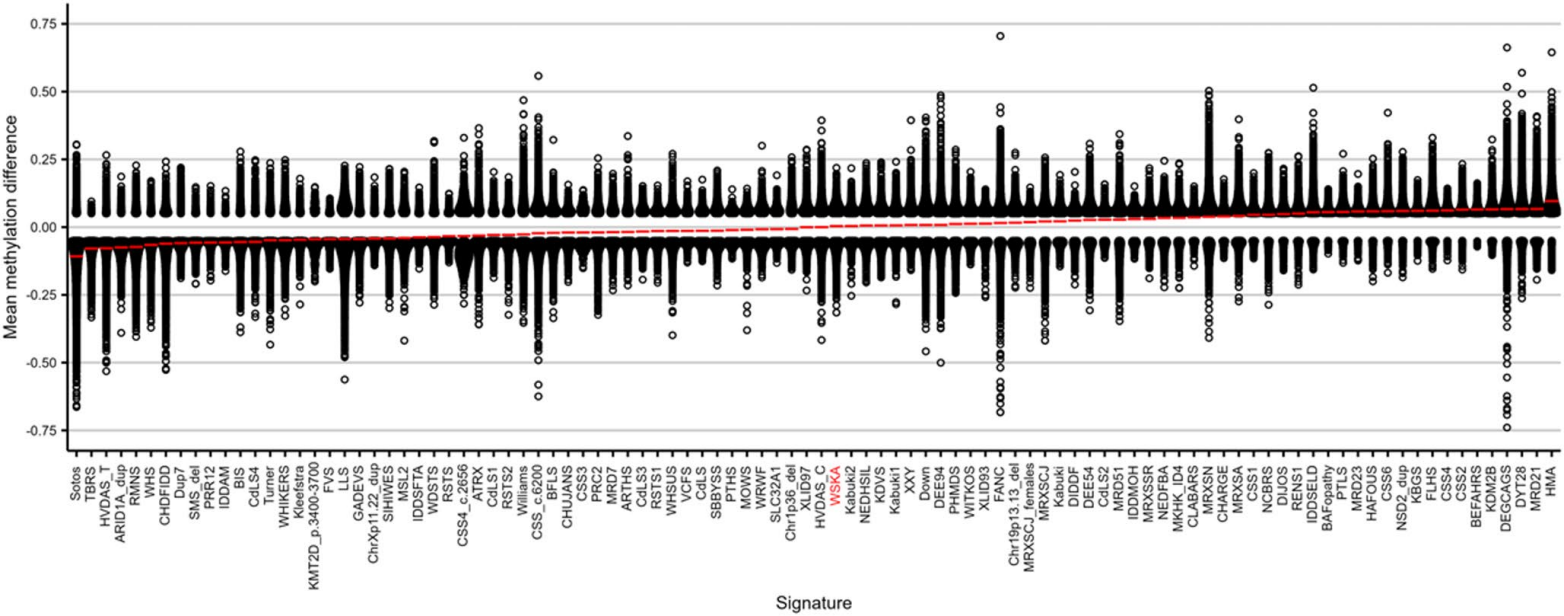
Global mean methylation of differentially methylated probes of WSKA cohort and other cohorts with known episignatures. The methylation difference of differentially methylated probes (DMPs) for each cohort is presented, sorted by the mean methylation. Each circle represents a probe, with red lines indicating the average methylation level

**Fig. 5:**
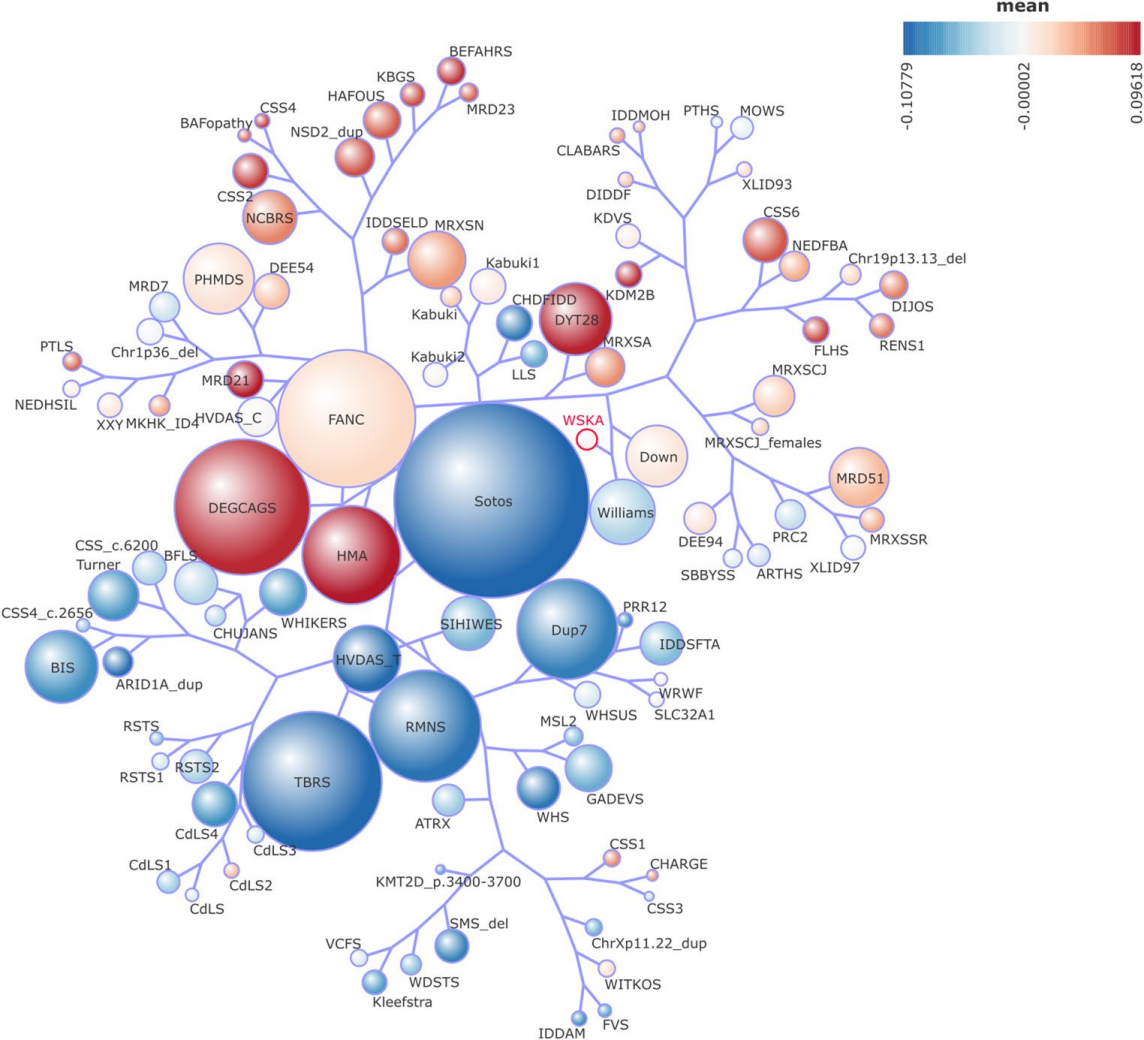
Comparison of WKSA cohort differentially methylated probes with other disorders with developed episignatures. The Tree and Leaf visualization demonstrates the Euclidean Clustering, with most similar cohorts on the same branch, for all cohorts using the top 500 DMPs per cohort. Cohort samples were aggregated by calculating the median value of each probe within a cohort. Leaf nodes represent individual cohorts, with node sizes reflecting the number of selected DMPs, and node colours indicating differences in the global mean methylation

Next, the mean methylation changes of all DMPs in the WSKA cohort compared to other disorder cohorts was investigated. By calculating the mean beta-values of all DMPs within each cohort, the overall methylation tendency, specifically hypo- or hypermethylation, was determined. The WSKA cohort exhibited a balanced profile characterized by a similar number of genome-wide probes that were hypermethylated and hypomethylated, with robustness of methylation differences similar in both directions (Fig. 4). To further explore the genomic methylation similarities between the WSKA cohort and other episignature disorders, a clustering analysis was performed. The top 500 DMPs were used for cohorts with sufficient data, while all available DMPs were utilized for cohorts with fewer than 500 DMPs. The tree and leaf diagram using these probes revealed a similarity between WSKA and Down syndrome (OMIM# 190685; caused by trisomy 21) and Williams syndrome (OMIM# 194050, caused by Chr7q11.23 deletions), which all clustered on the same branch (Fig. 5).

The Gene Ontology (GO) enrichment analysis of the WSKA DMPs unveiled 101 significant terms (adjusted p-value < 0.05) (Supplemental Table 2). These GO terms include many neuronal developmental and functional terms in all three ontology categories (biological process; BP, cellular component; CC, and molecular function; MF), as well as terms related to cell adhesion and G-protein couple receptor activity. In addition to DMPs, differentially methylated regions (DMRs), defined as ≥ 5 CpGs with ≥ 10% methylation change within 1 kb of each other, were also identified for the WSKA cohort. Fifty-one DMRs were identified, with 56.9% hypomethylated and 43.1% hypermethylated (Supplemental Table 3). 43% of the DMRs were within 1 kb of a promotor, indicating potential impacts on expression. When completing GO analysis for the identified DMRs, 5 terms were identified that primarily related to cell adhesion and plasma membrane adhesion (Supplemental Table 4).

## Discussion

While there are several studies that discuss overlapping clinical findings in patients with loss of function *ZNF462 *variants, there is still considerable phenotypic variability observed, even within family members carrying the same variant (Weiss et al. 2017; van der Laan et al. 2024; Kruszka et al. 2019; Huai et al. 2025). There is also little known about the natural history of WSKA, further increasing the challenge of identifying the condition clinically, especially in older individuals, highlighting uncertainties in prognosis (van der Laan et al. 2024; Kruszka et al. 2019). In addition to the obstacles described above, the rarity of the condition (thus far only 47 cases described in the literature) and the lack of characterized genotypic and phenotypic correlations complicate interpretation of VUSs (Weiss et al. 2017; Kruszka et al. 2019; Kerkhof et al. 2024; Hahne and Kuster 2012; Trajkova et al. 2024). When looking beyond the genetic sequence, to the epigenome, DNAm episignatures can serve as functional biomarkers for specific neurodevelopmental disorders, especially those for which the causative gene is part of the epigenetic machinery.

*ZNF462 *encodes Zinc Finger Protein 462, which is part of the zinc finger protein family who contribute to the regulation of gene expression and chromatin remodelling (Weiss et al. 2017; Kruszka et al. 2019; Hahne and Kuster 2012). Investigating its murine homolog suggests that*ZNF462* is essential for embryonic development through epigenetic silencing, and loss of function of *ZNF462* results in increased chromatin accessibility and subsequent ectopic expression of inappropriate lineage genes. Given the role of *ZNF462* in epigenetic regulation, there is rationale to explore an episignature for WSKA, a condition caused by *ZNF462 *haploinsufficiency. Previous studies have demonstrated the utility of episignatures in both unresolved cases (those with negative episignature findings and VUSs), those with an ambiguous clinical presentation, and screening of cases with limited genetic testing completed (Kerkhof et al. 2024; Levy et al. 2022; Trajkova et al. 2024; Aref-Eshghi et al. 2019; Sadikovic et al. 2021).

In this study we identified a unique DNA methylation episignature that successfully detected a previously described positive control/validation sample (Case 9) that carries a likely pathogenic *ZNF462* variant (Fig. 1) (van der Laan et al. 2024). This validation of the newly defined episignature, in conjunction with the leave-one-out cross validation findings, supports the reliability of the probe set identified as specific to patients carrying likely pathogenic or pathogenic loss of function *ZNF462 *variants that cause WSKA. We also tested a patient carrying a VUS who presents with features consistent with WSKA, including global developmental delay, coordination disorder, ASD, history of muscular Ventricular Septal Defect (VSD) with peripheral pulmonary stenosis, right preauricular tag and right corneal opacity. Interestingly, this patient did not match the identified episignature. This may be due to the fact that the VUS is a missense variant, which may result as a hypomorphic protein, whereas the identified episignature is primarily driven by loss-of-function variants. These observations highlight that missense variants may produce an atypical epigenetic response and episignature assessment should be interpreted in the context of variant type and clinical presentation. It is important to note DNAm findings alone should not be interpreted as evidence against pathogenicity as the number of episignatures, and signatures based on genotype or phenotype, is continually expanding; correlation with relevant clinical assessments and findings must be considered (Kerkhof et al. 2024). Indeed, previous work has demonstrated that episignatures are not only gene specific, but can also be specific to gene domains, gene regions, and even variant specific, as well as gene dosage specific (Levy et al. 2022, 2021; Haghshenas et al. 2024; Bend et al. 2019). Furthermore, other defined episignatures may be specific to loss of function variants, as episignatures for both Witteveen-Kolk syndromes (caused by heterozygous (likely) pathogenic *SIN3A* variants) and Developmental delay with variable intellectual disability and dysmorphic facies (caused by heterozygous (likely) pathogenic *JARID2 *variants) demonstrated specificity for loss of function variants and missense variants did not match (Coenen-van der Spek et al. 2023; Verberne et al. 2022). This finding for the VUS WSKA case, when considered with the broader literature, suggests that there may be a separate signature for hypomorphic *ZNF462* variants, but more cases are required to complete this investigation.

We next assessed genome-wide methylation changes for WSKA patients, beyond the probe set that was identified to be used as episignature. We assessed all DMPs and completed functional annotation as well as comparative analysis with all other conditions present on the EpiSign version 5 classifier (Kerkhof et al. 2024; Levy et al. 2022). When comparing the percentage of all overlapping DMPs between WSKA and other conditions, the top 4 conditions that demonstrate most overlap are also part of the epigenetic machinery, impacting histone methyltransferase *NSD1* (Sotos and HMA), linker histone *HIST1H1E* (RNMS), and transcription factor *ADNP* (HVDAS) (Fig. 2). *NSD1* haploinsufficiency causes Sotos syndrome whereas the duplication of the gene is thought to significantly contribute to the HMA phenotype caused by Chr5q35-qter duplications. The two syndromes demonstrate opposing clinical presentations stemming from opposite gene dosage changes in *NSD1 *(Hunter et al. 2005). *NSD1 *is also critical for proper embryonic development, demonstrating an essential role in the differentiation of the three primary germ layers, particularly the meso-endodermal cell lineages, through epigenetic regulation of histone methylation (Li et al. 2025; Tauchmann and Schwaller 2021). *NSD1 *targets putative distal enhancers to keep them in a primed state that can readily respond to differentiation signals (Li et al. 2025). Furthermore, histone methylation can crosstalk with DNAm, and dysregulation of *NSD1 *can disrupt DNA methylation (Tauchmann and Schwaller 2021). Figure 5 shows that both Sotos and HMA have many DMPs based on the size of the nodes within the tree and leaf plot. Given that *ZNF462* is also involved in cell differentiation, specifically silencing mesoendodermal genes, and the sheer number of DMPs impacted in Sotos and HMA, it is not surprising that all three conditions result in differential methylation at similar locations across the genome. When considering DMP overlap of WSKA with RMNS and HVDAS, this functional connection may be that all three genes impact heterochromatin protein 1 (HP1) recruitment. *ZNF462 *recruits a histone methyltransferase complex called GP9/GLP (also known as euchromatic histone methyltransferase 2 (EHMT2) and euchromatic histone methyltransferase 1) to deposit heterochromatin (Yelagandula et al. 2023). Loss of GP9 or GLP results in both loss of DNA methylation and HP1 binding (Collins and Cheng 2010; Tachibana et al. 2008, 2005). HP1 directly binds HIST1H1E, also known has histone 1.4, when it is methylated on H3K9 (a methylation histone mark deposited by *ZNF462*-recruited G9a/GLP complex) (Tachibana et al. 2005), promoting heterochromatin formation. Finally,*ANDP*is part of a chromatin remodelling complex called ChAHP, which includes CHD4 and HP1, to repress gene expression, especially during neurodevelopment (Clémot-Dupont et al. 2025; Ostapcuk et al. 2018). Given all three disorders impact heterochromatin formation during development through recruitment or direct interactions with HP1, it is plausible that many common impacts to the methylome would exist.

When assessing the similarities of only the top 500 DMPs in each episignature condition, meaning those CpG sites with most significant differential methylation,, the conditions most similar to WSKA are Williams-Beuren syndrome and Down syndrome (Fig. 5). In the Down syndrome region, overexpression of *DYRK1A*, a kinase that is thought to interact with chromatin opening SWI/SNF complex, disrupts a key regulator of neuronal differentiation and pluripotency, impacting all embryonic cell lineages (Lepagnol-Bestel et al. 2009; Canzonetta et al. 2008). The deletion on chromosome 7 that occurs in Williams-Beuren syndrome affects approximately 28 genes (depending on size), and haploinsufficiency of one of these genes, chromatin remodeler *BAZ1B*, causes widespread gene expression changes that alter neuronal differentiation (Lalli et al. 2016). The important roles that theses genes play in neuronal differentiation and gene regulation via epigenetic machinery may explain the functional overlap observed with *ZNF462*. When assessing the overlap of all identified DMPs, the WSKA cohort shared similarities with several distinct conditions; however, these relationships differed from the similarities observed when restricting the analysis to the top, most significantly methylated probes. Nonetheless, a common thread across all these cohorts is that the underlying pathologies stem from defects in chromatin remodeling.

When reviewing the GO analysis of both DMPs and DMRs, there are many pathways related to neuronal function and development, which is in-keeping with the neurodevelopmental presentation of WSKA. Additionally, cell adhesion-related terms were prominent in both DMP and DMRs GO analyses. Cell adhesion molecules play critical roles in neural circuit assembly, spatial organizations of axons and synaptic plasticity (Moreland and Poulain 2022; Dalva et al. 2007). Given that most WKSA patients present with developmental delay, the locations of the identified differential methylation suggest effects on genes important for neurodevelopment, however, functional studies such as gene expression analyses are required to better understand the consequences of these epigenetic changes. Finally, determination of identified DMR locations showed that 24 of the 51 DMRs fall within 3000 bp of a transcription start site (TSS), with 22 within 1000 bp of a TSS. When assessing the list of genes related to these TSSs, we see that they fall into categories that complement the GO analysis: Cell adhesion and structural integrity genes (*NECTIN4*,* PKP3*,* PCDHB7*,* FSCN2*,* EGFLAM*) and ion channels in the brain (*KCNN3*,* CHRNA4*) (Suppl Table 3). Genes required for proper embryonic development (*FIBIN*,* KHDC3L*) were also noted. Further exploration into expression of these genes in WSKA patients is required to better understand the significance of these differentially methylated regions and whether they contribute to WSKA phenotype. The observed differential methylation in our WKSA cohort may be due to the direct loss of ZNF462, the downstream impacts to interacting epigenetic machinery, or both. To delineate the functional impacts of *ZNF462* haploinsufficiency, additional studies with expanded datasets, such as whole-genome methylation data and protein-binding information, are required to correlate methylation changes with ZNF462 genomic and protein complex interactions.

A limitation of this study is the relatively small cohort size, which reflects the rarity of WSKA and limited sample availability. Nevertheless, the strong separation observed between cases and controls, reproducibility across leave-one-out analyses, and successful validation of an independent case support the robustness of the identified episignature.

## Conclusion

In this study, we identified a sensitive and specific DNAm episignature for patients with WSKA caused by pathogenic *ZNF462* variants. This episignature represents a powerful diagnostic biomarker that can distinguish WSKA from other neurodevelopmental disorders, providing insight into the epigenomic consequences of *ZNF462* haploinsufficiency. Comparative analyses highlight shared methylation impacts with other epigenetic machinery-related disorders that impact accessibility to chromatin during development, reflecting the role of disrupted chromatin regulation in disease pathology. Our findings underline the importance of episignatures in refining the clinical diagnosis and interpretation of variants. The episignature for WSKA can be included in the multiclass EpiSign classifier, for screening patients with suspected rare neurodevelopmental disorders, particularly those with ambiguous phenotypes, thereby facilitating differential diagnosis and potentially shortening the often lengthy diagnostic odyssey.

## Supplementary Information

Below is the link to the electronic supplementary material.

Supplementary Material 1

Supplementary Material 2 Figure S1. Leave-one-out cross-validation results for discovery training cases. In each cross-validation set, a single test case sample (represented in dark blue) from original training cohort was utilized for testing, while the remaining WSKA training cases (denoted in red) were used to train the episignature against the matched controls (represented in light blue). The case left out is identified above each set of LOOCV plots. Clustering of the “left out” or testing cases was assessed by Euclidean hierarchical clustering and MDS plots. MVP scores of the testing case against the trained SVM were calculated to assess concordance. The SVM was trained using the selected the remaining training WSKA training cases, as well as 75% of controls and other EpiSignTM samples (shown in blue). The remaining 25% of controls and EpiSignTM other disorder samples were reserved for testing, along with the WSKA left out or testing case (shown in gray). (PDF 4207 kb)

## Data Availability

The individual genomic and epigenomic or any other personally identifiable data for other samples in the EpiSign Knowledge Database (EKD) are not available for deposition in publicly accessible databases due to institutional and ethics restrictions. Specifically, these include data and samples submitted from external institutions to London Health Sciences EKD that are subject to Institutional Material and Data Transfer agreements, data submitted to London Health Sciences for episignature assessment under Research Services Agreements, and research study cohorts under Institutional Research Ethics Approval (Western University REB 106302; and REB 116108). Some of the software packages used in this study are publicly available as described in the Materials and Methods. EpiSign^TM^ is a commercial software and is not publicly available.
