# Supplementary figures and images for: Discovery of a DNA methylation episignature for Weiss-Kruszka syndrome
Source: Hum Genet. 2026 Jul 20;145(1):63. doi: 10.1007/s00439-026-02846-1 (PMC13385256; doi:10.1007/s00439-026-02846-1)

Supplemental Figure 1

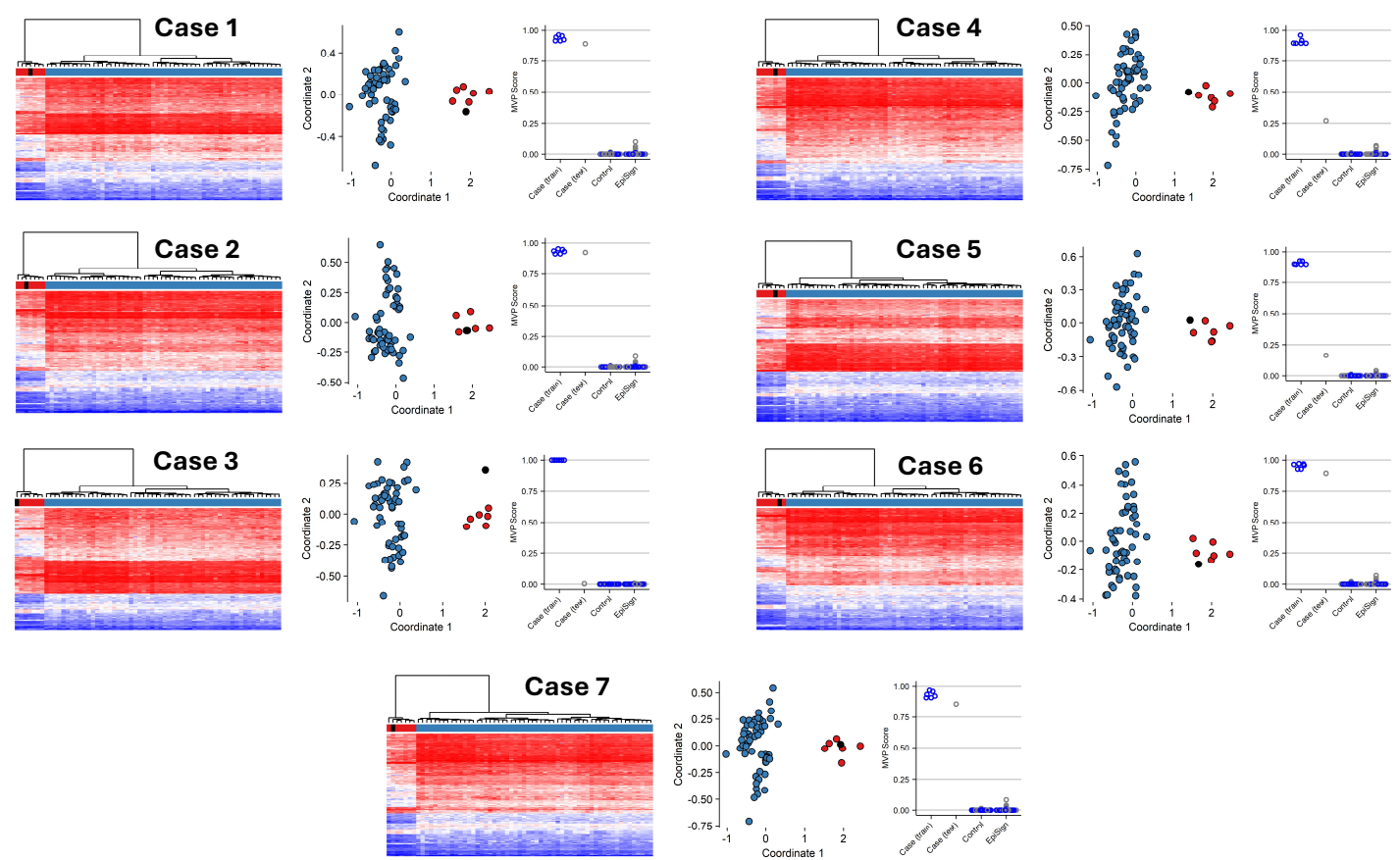

Supplement: Supplementary file 2 — Supplementary Material 2 Figure S1. Leave-one-out cross-validation results for discovery training cases. In each cross-validation set, a single test case sample (represented in dark blue) from original training cohort was utilized for testing, while the remaining WSKA training cases (denoted in red) were used to train the episignature against the matched controls (represented in light blue). The case left out is identified above each set of LOOCV plots. Clustering of the “left out” or testing cases was assessed by Euclidean hierarchical clustering and MDS plots. MVP scores of the testing case against the trained SVM were calculated to assess concordance. The SVM was trained using the selected the remaining training WSKA training cases, as well as 75% of controls and other EpiSignTM samples (shown in blue). The remaining 25% of controls and EpiSignTM other disorder samples were reserved for testing, along with the WSKA left out or testing case (shown in gray). (PDF 4207 kb) [file 439_2026_2846_MOESM2_ESM.pdf]
